# Resveratrol enhances anticancer effects of paclitaxel in HepG2 human liver cancer cells

**DOI:** 10.1186/s12906-017-1956-0

**Published:** 2017-10-04

**Authors:** Qin Jiang, Manyi Yang, Zhan Qu, Jixiang Zhou, Qi Zhang

**Affiliations:** 1 Department of Hepatobiliary & Pancreatic Surgery, Xiangya Hospital, Central South University, Changsha, 410008 China; 2 Department of Ultrasonography, Xiangya Hospital, Central South University, Changsha, 410008 China; 3 National Hepatobiliary & Enteric Surgery Research Center, Xiangya Hospital, Central South University, Changsha, 410008 China

**Keywords:** Resveratrol, Paclitaxel, HepG2 human liver cancer cells, Sensitizing, Apoptosis

## Abstract

**Background:**

The aim of this in vitro study was to measure the enhanced anticancer effects of Res (resveratrol) on PA (paclitaxel) in HepG2 human liver cancer cells.

**Methods:**

The MTT (thiazolyl blue tetrazolium bromide, 3-(4,5-Dimethylthiazol-2-yl)-2,5-Diphenyltetrazolium Bromide), flow cytometry, qPCR (real-time quantitative polymerase chain reaction) and western blot assay were used for cells growth inhibitory effects, cells apoptosis (DNA content of sub-G1), mRNA and protein expressions, respectively.

**Results:**

The 10 μg/mL of Res had no growth inhibitory effect on Nthy-ori 3–1 normal cells or HepG2 cancer cells meanwhile the 5 or 10 μg/mL of PA also had no growth inhibitory effect on Nthy-ori 3–1 normal cells. Where as PA-L (5 μg/mL) and PA-H (10 μg/mL) had the growth inhibitory effects in HepG2 cancer cells, and Res increase these growth inhibitory effects. By flow cytometry experiment, after Res (5 μg/mL) + PA-H (10 μg/mL) treatment, the HepG2 cells showed the most apoptosis in cells as compared to other treatments groups, and after additionally treated with Res, both the apoptosis cells of two concentrations PA were raised. As PA raised it also raised the mRNA and protein expressions of caspase-3, caspase-8, caspase-9, Bax (Bcl-2 assaciated X protein), p53, p21, IκB-α (inhibitor of NF-κB alpha), Fas (factor associated suicide), FasL (factor associated suicide ligand), TIMP-1 (tissue inhibitor of metalloproteinases 1), TIMP-2 (tissue inhibitor of metalloproteinases 2) and decrease Bcl-2 (B cell leukemia 2), Bcl-xL (B cell leukemia extra large), HIAP-1 (cIAP-1, cellular inhibitor of apoptosis 1), HIAP-2 (cIAP-2, cellular inhibitor of apoptosis 2), NF-κB (nuclear factor kappa B), COX-2 (cyclooxygenase 2), iNOS (inducible nitric oxide synthase), MMP-2 (metalloproteinase 2), MMP-9 (metalloproteinase 9), EGF (epidermal growth factor), EGFR (epidermal growth factor receptor), VEGF (vascular endothelial growth factor), Fit-1 (VEGFR-1, vascular endothelial growth factor receptor 1). Meanwhile, the 5 μg/mL of Res could enhance these mRNA expressions changes as compared to the control cells.

**Conclusion:**

From these results, we can conclude that Res could raise the anticancer effects of PA in HepG2 cells, Res could be used as a good sensitizing agent for PA.

## Background

Paclitaxel, the chemical substance which was isolated from the bark and trunk of Yew Pacific, can disturb the dynamic equilibrium of tubulin and its -microtubule dimer, it contribute to induce and promote tubulin polymerization and assembly, also prevents its depolymerization, enhancing the stability of tubulin, which ultimately helps in inhibiting the growth of cancer cells [1]. Paclitaxel inhibits the dynamic regeneration of microtubule network which is the normal process of mitosis, more precisely prevents the formation of mitotic spindle, breaking chromosome and inhibiting proliferation and migration [2]. Research has showed that in patients with acute myeloblastic or lymphoblastic leukemia, the ratio of monocytes with DNA chain segment which apoptosis rises from 0.4%–16% to 3.4%–45% after being treated with paclitaxel [3]. When incubating with paclitaxel, human cancer cell lines also were apoptosis. The ratio of human hepatocellular cell lines or breast cancer cell lines which apoptosis rises with the increase of the concentration of paclitaxel. Further studies also showed that paclitaxel could also regulate the body’s immune function by having the interaction with macrophages in order to decrease the release of TNF-α and the number of its receptors. It also promotes the release of interleukin-1, IFN-α (interferon alpha) and IFN-β (interferon beta) to kill or inhibit cancer cells [4].

Study reported written by experts and scholars as well as clinical application showed that paclitaxel not only had a good curative effect on ovarian cancer, uterine cancer and breast cancer, but also significantly helped in curing pancreatic cancer, colon cancer, prostate cancer, metastatic renal carcinoma, acute pancreatitis, retina tumor, malignant melanoma tumors, head and neck cancer as well as other cancers [5]. Clinical trials revealed that paclitaxel could treat other diseases to some extent, it fights against rheumatoid arthritis, malaria, and also improves stroke, Alzheimer’s disease and congenital polycystic kidney disease [6].

Resveratrol is a kind of polyphenol compound, mainly originating from peanut, red wine, *Polygonum cuspidatum*, mulberry and other plants [7–13]. Resveratrol is a natural polyphenol with extensive biological functions, can also reduce the platelet aggregation, prevent and treat atherosclerosis, cardiovascular diseases, cerebrovascular diseases and most importantly act as a cancer chemopreventive agent [14]. Kim et al. [15] had found that resveratrol could change cell cycle, and influence the expression of apoptosis-related genes. In-depth research found that resveratrol could enhance the sensitizing effect of prostate cancer cell line PC-3 in vitro [16].

Cancer inhibitors exist in various plants naturally, and have very good effects on human cancer prevention. These cancer inhibitors that occur naturally have low toxicity, as well as can reduce the pain of patients during the treatment [17]. But the activity of many cancer inhibitors existing in natural plants is lower than that of synthetic drugs, and the combination of different natural cancer inhibitors can substantially improves the treatment effect of cancer, so finding out a reasonable combination becomes the most important thing to improve and enhance the anti-cancer effects resources that occurs naturally.

Due to its unique anticancer mechanism, paclitaxel has accepted the recognition among the medical expertise and oncology related industries. However, paclitaxel is insoluble in water and has clinical toxicity and side effects, which affects its application. In recent years, people begin to study and explore the medicinal preparation of paclitaxel, aiming to find a breakthrough to overcome the above difficulties. In this paper, we study the effects of paclitaxel combined with resveratrol, to reduce the concentration of treatment effects of paclitaxel and resveratrol, Meanwhile observe their joint anticancer effects.

## Methods

### Cancer cells

Human normal liver cell lines L02 and HepG2 human hepatoma cells were purchased from Conservation Genetics CAS Kunming Cell Bank (Kunming City, Yunnan Province, China).

### Cells culture

The L02 and HepG2 cells were cultured in DMEM medium (Gibco Co., Birmingham, MI, USA) added with 10% FBS (Gibco) and 1% penicillin-streptomycin (Gibco) at 37 °C in 5% CO_2_ incubator (MCO18AIC; SANYO, Tokyo, Japan). The medium was changed every 2 d.

### MTT assay

Culture solution was added to adjust the concentration of cancer cells in logarithmic growth phase to 2 × 10^4^/dish, which were added to the 96-well culture plate with 50 μL per hole, and placed in incubator with 5% CO_2_ at 37 °C for 24 h. Res (Sigma, St. Louis, MO, USA) after that PA were added into the 96-well plate with 50 μL per well, to adjust the concentration of cancer cells to 5 μg/mL Res adding 5 or 10 μg/mL PA eventually. The 50 μL culture solution was added to the blank control group, which was cultured in CO_2_ incubator (MCO18AIC; SANYO) for 48 h. Followed by, the blank control group which was added with MTT solution after the supernatant was removed and then incubated for 4 h. The 100 μL DMSO was added to the blank control group after the supernatant was removed and shocked for 30 min, the enzyme standard instrument were used to detect at 570 nm [18].

### Flow cytometry

Single cell suspension was centrifuged to remove stationary liquid and washed by 3 mL PBS twice, and then centrifuged for 5 min; added with 1 ml PI staining solution and incubated in refrigerator at 4 °C for 30 min without explosion to sunshine; and then filtered by 500-well copper mesh; flow cytometry (Accuri C6, BD, Franklin Lakes, NJ, China) detection and argon ion laser with 15 mA excitation light source and 488 nm wavelength were used for testing, and 630 nm band-pass filter to receive the light. The 1 × 10^4^ cells were collected by FSC/SSC scattered point diagram method, with gating technology used to exclude adhesive cells and cell debris, to analyze the percentage of apoptotic cells in PI fluorescence histogram [18].

### qPCR assay

RNAzol reagent was used to extract the total RNA from cancer cells, and DNase RNase-free was adopted to digest total RNA at 37 °C for 15 min, and then RNeasy kit to purify RNA to adjust its concentration to 1 μg/μL. RNA (2 μg) was used as the template to synthetize cDNA by reacting with reverse transcriptase at 37 °C for 120 min, at 99 °C for 4 min, and at 4 °C for 3 min respectively. After that, reverse transcription-polymerase chain reaction method was adopted to amplify the DNA expressions (Table 1), to measure the transcription level of mRNA, and GAPDH was used as the housekeeping genes of internal control group. Finally, agarose electrophoresis with 1% ethidium bromide was adopted to check PCR (ABI Q3, Thermo Fisher Scientific, Inc., Waltham, MA, USA) amplified products [19].

**Table 1:** Sequences of primers were used in this study

Gene name	Sequence
Caspase-3	Forward: 5′-CAA ACT TTT TCA GAG GGG ATC G-3′
	Reverse: 5′-GCA TAC TGT TTC AGC ATG GCA-3′
Caspase-8	Forward: 5′-CCC CAC CCT CAC TTT GCT-3′
	Reverse: 5′-GGA GGA CCA GGC TCA CTT A-3′
Caspase-9	Forward: 5′-GGC CCT TCC TCG CTT CAT CTC-3′
	Reverse: 5′-GGT CCT TGG GCC TTC CTG GTA T-3′
Bax	Forward: 5′-AAG CTG AGC GAG TGT CTC CGG CG-3′
	Reverse: 5′-CAG ATG CCG GTT CAG GTA CTC AGT C-3′
Bcl-2	Forward: 5′-CTC GTC GCT ACC GTC GTG ACT TGG-3′
	Reverse: 5′-CAG ATG CCG GTT CAG GTA CTC AGT C-3′
Bcl-xL	Forward: 5′-CCC AGA AAG GAT ACA GCT GG-3′
	Reverse: 5′-GCG ATC CGA CTC ACC AAT AC-3′
p53	Forward: 5′-GCT CTG ACT GTA CCA CCA TCC-3′
	Reverse: 5′-CTC TCG GAA CAT CTC GAA GCG-3′
p21	Forward: 5′-CTC AGA GGA GGC GCC ATG-3′
	Reverse: 5′-GGG CGG ATT AGG GCT TCC-3′
NF-κB	Forward: 5′-CAC TTA TGG ACA ACT ATG AGG TCT CTG G-3′
	Reverse: 5′-CTG TCT TGT GGA CAA CGC AGT GGA ATT TTA GG-3′
IκB-α	Forward: 5′-GCT GAA GAA GGA GCG GCT ACT-3′
	Reverse: 5′-TCG TAC TCC TCG TCT TTC ATG GA-3′
Fas	Forward: 5′-GAA ATG AAA TCC AAA GCT-3′
	Reverse: 5′-TAA TTT AGA GGC AAA GTG GC-3′
FasL	Forward: 5′-GGA TTG GGC CTG GGG ATG TTT CA-3′
	Reverse: 5′-TTG TGG CTC AGG GGC AGG TTG TTG-3′
TIMP-1	Forward: 5′-GTC AGT GAG AAG CAA GTC GA-3′
	Reverse: 5′-ATG TTC TTC TCT GTG ACC CA-3′
TIMP-2	Forward: 5′-TGG GGA CAC CAG AAG TCA AC-3′
	Reverse: 5′-TTT TCA GAG CCT TGG AGG AG-3′
MMP-2	Reverse: 5′-CTT CTT CAA GGA CCG GTT CA-3′
	Forward: 5′-GCT GGC TGA GTA CCA GTA-3′
MMP-9	Reverse: 5′-TGG GCT ACG TGA CCT ATG AC-3′
	Forward: 5′-GCC CAG CCC ACC TCC ACT CC-3′
HIAP-1	Reverse: 5′-GCC TGA TGC TGG ATA ACT GG-3′
	Forward: 5′-GGC GAC AGA AAA GTC AAT GG-3′
HIAP-2	Reverse: 5′-GCC TGA TGC TGG ATA ACT GG-3′
	Forward: 5′-GCT CTT GCC AAT TCT GAT GG-3′
COX-2	Reverse: 5′-TTA AAA TGA GAT TGT CCG AA-3′
	Forward: 5′-AGA TCA CCT CTG CCT GAG TA-3′
iNOS	Reverse: 5′-AGA GAG ATC GGG TTC ACA-3′
	Forward: 5′-CAC AGA ACT GAG GGT ACA-3′
EGF	Reverse: 5′-GCC AAG CTC AGA AGG CTA C-3′
	Forward: 5′-CAG GCC AGC CTC GTC TCA T-3′
EGFR	Reverse: 5′-TCG GTG CTG TGC GAT TTA-3′
	Forward: 5′-TTT CTG GCA GTT GCT CCT C-3′
VEGF	Reverse: 5′-GCA CCC ATG GCA GAA GGA GGA G-3′
	Forward: 5′-GTG CTG ACG CTA ACT GAC C-3′
Fit-1	Reverse: 5′-CAA GTG GCCAGA GGC ATG GAG TT-3′
	Forward: 5′-GAT GTA GTC TTTACC ATC CTG TTG-3′
GAPDH	Reverse: 5′-CGG AGT CAA CGG ATT TGG TC-3′
	Forward: 5′-AGC CTT CTC CAT GGT CGT GA-3′

### Western blot assay

When the cells of each group finished culturing, protein lysates was added to get total protein extracts. Bradford method was adopted to determine the concentration of proteins. The 10% separation gel and 5% stacking gel were prepared for SDS-PAGE electrophoresis and trarsmembrane. The 5% nonfat milk sealing liquid was used to seal each group for 2 h, and then added with diluted primary antibody (at 4 °C overnight). Each group was added with second antibody after being washed by TBST for 3 times, incubated by shocking at room temperature for 2 h, and then rinsed by TBST for 3 times. Afterwards, ECL coloration method was used to develop the cells of each group (6600, Tanon, Shanghai, China), and GIS gel image to analyze system and processing [20].

### Statistical analysis

The in vitro experiments were presented as mean ± standard deviation (SD). Differences between the mean values for individual groups were assessed with one-way analysis of variance (ANOVA) with Dunnett’s post-hoc test. Using SPSS 22.0 (IBM, New York, NY, USA.

## Results

### Growth inhibitory effects of Res and PA in Nthy-ori 3-1 and HepG2 cells

**Fig. 1:**
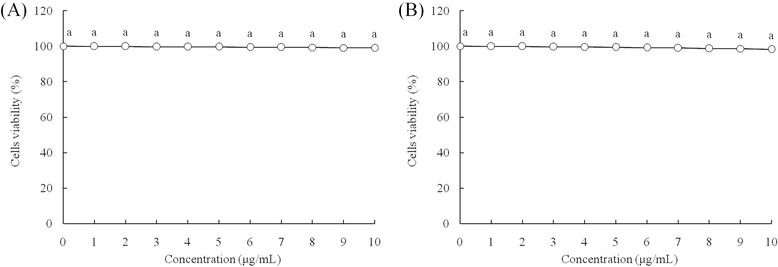
Effect of resveratrol (**a**) and paclitaxel (**b**) on the growth of human normal liver cell lines L02. ^a^ Mean values with different letters over the points are no significantly different (*P* < 0.05) according to Dunnett’s post-hoc test

**Fig. 2:**
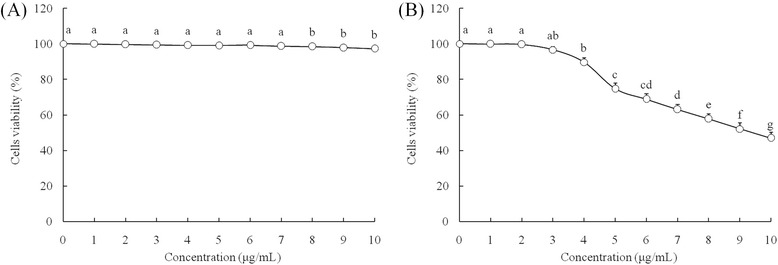
Effect of resveratrol (**a**) and paclitaxel (**b**) on the growth of HepG2 human liver cancer cells. ^a-g^ Mean values with different letters over the points are significantly different (*P* < 0.05) according to Dunnett’s post-hoc test

After 0–10 μl/mL of Res and PA treatment, Res and PA could not inhibit the growth of Nthy-ori 3–1 cells (Fig. 1), and Res also could not inhibit the growth of HepG2 cells (Fig. 2), but PA could inhibit the growth of HepG2 cells above 5 μl/mL concentration. Base on these results, 10 μl/mL of Res, 5 and 10 μl/mL of PA were chosen for further experiment to get the promising results. By the MTT assay, the untreated HepG2 cells showed the OD_540_ value at 0.431 (Table 2), after Res or PA treatment, the OD_540_ values were reduced. Adding Res treatment, the HepG2 cells growth inhibitory effects was higher than only PA treatment.

**Table 2:** Growth inhibitory effects of HepG2 human liver cancer cells by resveratrol and paclitaxel by an MTT

Treatment	OD_570_ value	Inhibitory rate (%)
Control	0.431 ± 0.003^a^	/
PA-L	0.322 ± 0.006^b^	25.3 ± 2.1^d^
Res + PA-L	0.287 ± 0.008^c^	33.4 ± 2.2^c^
PA-H	0.202 ± 0.008^d^	53.1 ± 2.3^b^
Res + PA-H	0.089 ± 0.005^e^	79.4 ± 1.9^a^

*PA-L* 5 μg/mL of paclitaxel, *Res + PA-L* 10 μg/mL of resveratrol +5 μg/mL of paclitaxel, *PA-H* 10 μg/mL of paclitaxel, *Res + PA-H* 10 μg/mL of resveratrol +10 μg/mL of paclitaxel

^a-e^Mean values with different letters in the same column are significantly different (*P* < 0.05) according to Dunnett’s post-hoc test

### DNA content of sub-G1 HepG2 cells

**Fig. 3:**
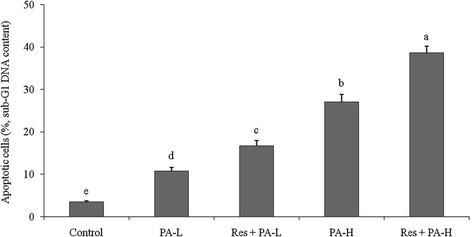
Apoptosis inducing effects (DNA content of sub-G1) of resveratrol (Res) and paclitaxel (PA) in HepG2 human liver cancer cells. ^a-e^ Mean values with different letters over the bars are significantly different (*P* < 0.05) according to Dunnett’s post-hoc test. PA-L: 5 μg/mL of paclitaxel; Res + PA-L: 10 μg/mL of resveratrol +5 μg/mL of paclitaxel; PA-H: 10 μg/mL of paclitaxel; Res + PA-H: 10 μg/mL of resveratrol +10 μg/mL of paclitaxel

The flow cytometry showed that control cells has only 3.5 ± 0.3% DNA content of sub-G1 of HepG2 cells (apoptotic cells), and as per the results in the other groups the cells have more apoptosis because of PA or Res + PA treatment (Fig. 3). PA-L, Res + PA-L, PA-H and Res + PA-H group had 10.8 ± 0.8%, 16.7 ± 1.3%, 27.1 ± 1.8% and 38.6 ± 1.7% apoptosis of HepG2 cells, respectively.

### mRNA and protein expressions of caspases

**Fig. 4:**
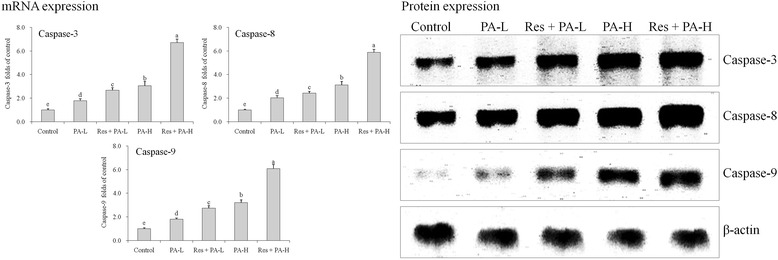
The mRNA and protein expression of caspase-3 caspase-8 and caspase-9 in HepG2 human liver cancer cells. ^a-e^ Mean values with different letters over the bars are significantly different (*P* < 0.05) according to Dunnett’s post-hoc test. PA-L: 5 μg/mL of paclitaxel; Res + PA-L: 10 μg/mL of resveratrol +5 μg/mL of paclitaxel; PA-H: 10 μg/mL of paclitaxel; Res + PA-H: 10 μg/mL of resveratrol +10 μg/mL of paclitaxel

By qRCR and western blot experiments, control cells showed the weakest caspase-3, caspase-8 and caspase-9 also the mRNA and protein expressions were weakest too (Fig. 4). PA treated cells showed the remarkably stronger caspase-3, caspase-8 and caspase-9 expressions than control cells, Res adding treatment hike up these expressions significantly and Res + PA-H had the strongest caspase-3, caspase-8 and caspase-9 expressions.

### mRNA and protein expressions of Bax, Bcl-2 and Bcl-xL

**Fig. 5:**
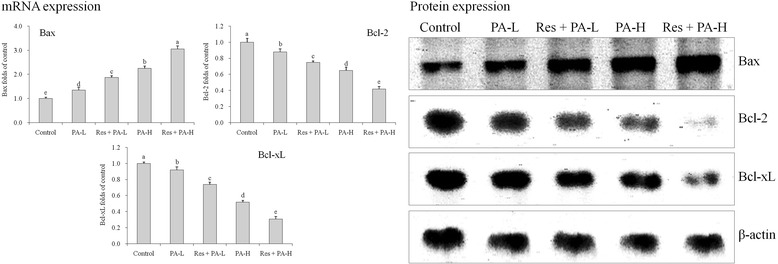
The mRNA and protein expression of Bax, Bcl-2 and Bcl-xL in HepG2 human liver cancer cells. ^a-e^ Mean values with different letters over the bars are significantly different (*P* < 0.05) according to Dunnett’s post-hoc test. PA-L: 5 μg/mL of paclitaxel; Res + PA-L: 10 μg/mL of resveratrol +5 μg/mL of paclitaxel; PA-H: 10 μg/mL of paclitaxel; Res + PA-H: 10 μg/mL of resveratrol +10 μg/mL of paclitaxel

PA treated HepG2 cells had the high Bax mRNA and protein expressions and low Bcl-2, Bcl-xL expressions (Fig. 5); where as Res + PA-H were showing the highest Bax expressions than other groups cells, but Res + PA-H showed the lowest Bcl-2, Bcl-xL expressions.

### mRNA and protein expressions of Fas and FasL

**Fig. 6:**
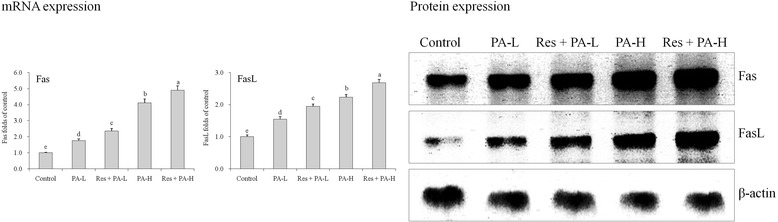
The mRNA and protein expression of Fas and FasL in HepG2 human liver cancer cells. ^a-e^ Mean values with different letters over the bars are significantly different (P < 0.05) according to Dunnett’s post-hoc test. PA-L: 5 μg/mL of paclitaxel; Res + PA-L: 10 μg/mL of resveratrol +5 μg/mL of paclitaxel; PA-H: 10 μg/mL of paclitaxel; Res + PA-H: 10 μg/mL of resveratrol +10 μg/mL of paclitaxel

The control cells had the lowest Fas and FasL mRNA and protein expressions (Fig. 6), after sample treatment, these expressions were elevated, adding Res treated cells had the remarkably higher Fas and FasL expressions with respect to only PA treated cells, and as we increase the concentration of the combination PA and Res expressions increase simultaneously.

### mRNA and protein expressions of p53 and p21

**Fig. 7:**
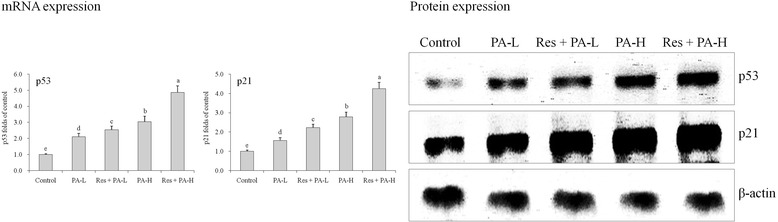
The mRNA and protein expression of p53 and p21 in HepG2 human liver cancer cells. ^a-e^ Mean values with different letters over the bars are significantly different (P < 0.05) according to Dunnett’s post-hoc test. PA-L: 5 μg/mL of paclitaxel; Res + PA-L: 10 μg/mL of resveratrol +5 μg/mL of paclitaxel; PA-H: 10 μg/mL of paclitaxel; Res + PA-H: 10 μg/mL of resveratrol +10 μg/mL of paclitaxel

The p53 and p21 mRNA and protein expressions of control cells were weakest (Fig. 7), PA could increase these expressions, and high concentration of PA had the stronger capability to increase p53 and p21 activities. The Res and PA combination showed the stronger p53 and p21 expressions than only PA treatment.

### mRNA and protein expressions of HIAP-1 and HIAP-2

**Fig. 8:**
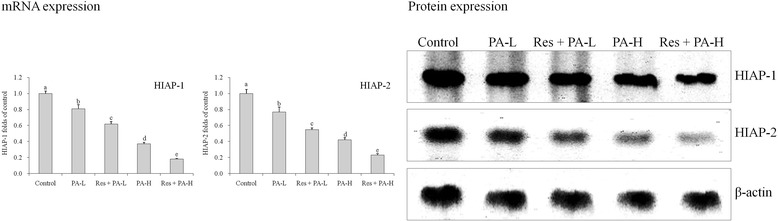
The mRNA and protein expression of HIAP-1 and HIAP-2 in HepG2 human liver cancer cells. ^a-e^ Mean values with different letters over the bars are significantly different (P < 0.05) according to Dunnett’s post-hoc test. PA-L: 5 μg/mL of paclitaxel; Res + PA-L: 10 μg/mL of resveratrol +5 μg/mL of paclitaxel; PA-H: 10 μg/mL of paclitaxel; Res + PA-H: 10 μg/mL of resveratrol +10 μg/mL of paclitaxel

The control cells had the strongest HIAP-1 and HIAP-2 mRNA and protein expressions (Fig. 8), PA substantially reduce the expression as compared to control cells, high concentration of PA and Res (Res + PA-H) showed the weakest HIAP-1 and HIAP-2 expressions among all groups.

### mRNA and protein expressions of NF-κB and IκB-α

**Fig. 9:**
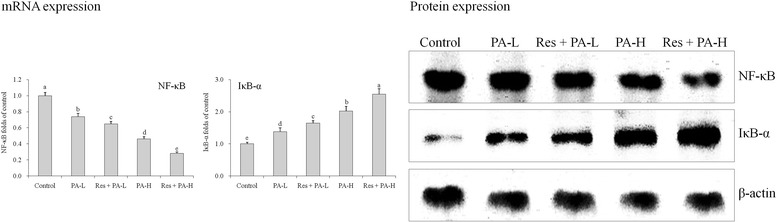
The mRNA and protein expression of NF-κB and IκB-α in HepG2 human liver cancer cells. ^a-e^ Mean values with different letters over the bars are significantly different (P < 0.05) according to Dunnett’s post-hoc test. PA-L: 5 μg/mL of paclitaxel; Res + PA-L: 10 μg/mL of resveratrol +5 μg/mL of paclitaxel; PA-H: 10 μg/mL of paclitaxel; Res + PA-H: 10 μg/mL of resveratrol +10 μg/mL of paclitaxel

Res + PA-H group cells had the lowest NF-κB mRNA and protein expressions and the highest IκB-α (Fig. 9)*.* These expression were higher than only PA-H treated group, this revealed that Res could increase these effects when present with PA.

### mRNA and protein expressions of COX-2 and iNOS

**Fig. 10:**
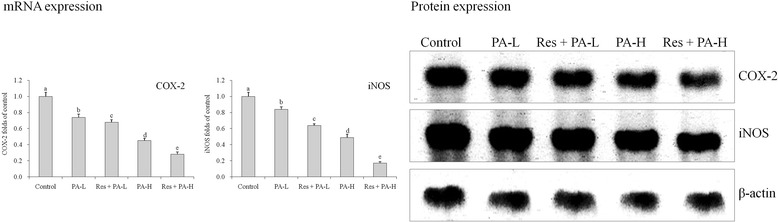
The mRNA and protein expression of COX-2 and iNOS in HepG2 human liver cancer cells. ^a-e^ Mean values with different letters over the bars are significantly different (*P* < 0.05) according to Dunnett’s post-hoc test. PA-L: 5 μg/mL of paclitaxel; Res + PA-L: 10 μg/mL of resveratrol +5 μg/mL of paclitaxel; PA-H: 10 μg/mL of paclitaxel; Res + PA-H: 10 μg/mL of resveratrol +10 μg/mL of paclitaxel

Control group cells showed the strongest COX-2 and iNOS mRNA and protein expressions, but Res + PA-H group cells showed the weakest expressions (Fig. 10)*.*

### mRNA and protein expressions of TIMP-1, TIMP-2, MMP-2 and MMP-9

**Fig. 11:**
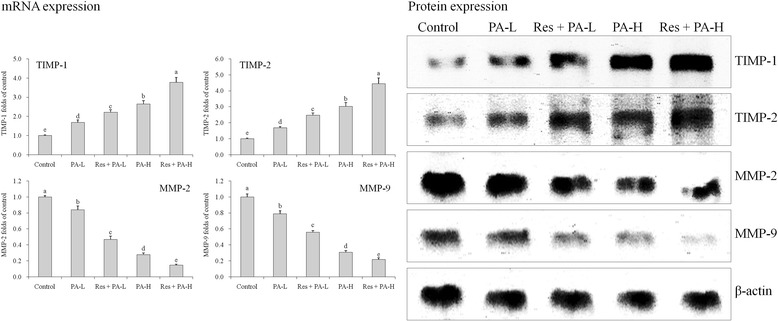
The mRNA and protein expression of TIMP-1, TIMP-2, MMP-2 and MMP-9 in HepG2 human liver cancer cells. ^a-e^ Mean values with different letters over the bars are significantly different (*P* < 0.05) according to Dunnett’s post-hoc test. PA-L: 5 μg/mL of paclitaxel; Res + PA-L: 10 μg/mL of resveratrol +5 μg/mL of paclitaxel; PA-H: 10 μg/mL of paclitaxel; Res + PA-H: 10 μg/mL of resveratrol +10 μg/mL of paclitaxel

The TIMP-1, TIMP-2 mRNA and protein expressions in control cells were lowest than other groups cells, but MMP-2, MMP-9 expressions were highest than other groups cells (Fig. 11)*.* PA could raise TIMP-1, TIMP-2 expressions and reduce MMP-2, MMP-9 expressions as compared to the control cells, and and after addition of Res it showed higher TIMP-1, TIMP-2 expressions and lower MMP-2, MMP-9 expressions than only PA treated cells.

### mRNA and protein expressions of EGF, EGFR, VEGF and Fit-1

**Fig. 12:**
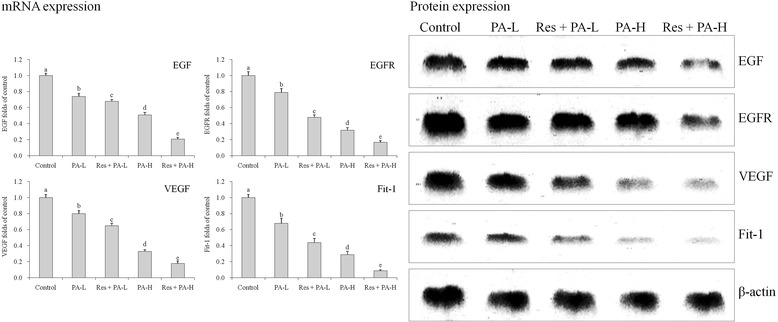
The mRNA and protein expression of EGF, EGFR, VEGF and Fit-1 in HepG2 human liver cancer cells. ^a-e^ Mean values with different letters over the bars are significantly different (*P* < 0.05) according to Dunnett’s post-hoc test. PA-L: 5 μg/mL of paclitaxel; Res + PA-L: 10 μg/mL of resveratrol +5 μg/mL of paclitaxel; PA-H: 10 μg/mL of paclitaxel; Res + PA-H: 10 μg/mL of resveratrol +10 μg/mL of paclitaxel

PA treatment reduce EGF, EGFR, VEGF, Fit-1 mRNA and protein expressions as compared to the control cells (Fig. 12), and high concentration PA showed further reduction in expression of EGF, EGFR, VEGF, Fit-1. After Res addition, the experiment proved that expression was lower than only treated with PA, Res + PA-H had the lowest EGF, EGFR, VEGF, Fit-1 expressions.

## Discussion

Apoptosis of Cancer cell plays an important role in the occurrence and development of cancer, Wong et al. [20] found that a lot of receptor-mediated cell signal transduction and many different genes are involved in the activation of cancer cells apoptosis, and regulation of cancer cell apoptosis respectively. As an upstream protein involved in exogenous apoptosis, caspase-8 shears and activates downstream apoptosis-inducing proteins such as caspase-3, caspase-6 and caspase-7, causing cell apoptosis [21]. Apaf-l can bond to the original structural domain of the precursor of caspase-9 through the complementary domain of caspase, leading to the self-activation of caspase-9, which further activates downstream caspase-3, caspase-6 and caspase-7, and ultimately inducing endogenous apoptosis of cells [22]. Caspase-3 involves both exogenous and endogenous apoptosis, and many apoptotic factors work on downstream effector caspase-3 ultimately to induce cell apoptosis [23]. In this study, PA could raised the mRNA and protein expressions of caspase-3, caspase-8 and caspase-9, Res raised these effects of Res treatment, this combination done a good cancer cells apoptosis effects.

The inhibition of apoptosis has a vital significance to the incidence and development of cancer, Proteins in Bcl-2 family play the important roles in regulating the apoptosis of cancer cells. Bcl-2 family is made up of apoptosis inhibitory factor (Bcl-2 and Bcl-xL) and apoptosis-promoting factor (Bax), their ratio determines whether the cell is able to accept the apoptotic signal or not [24]. To a certain extent, apoptosis or apoptosis inhibition are regulated by the above two genes. The disturbance of apoptosis regulation is crucial in the development of tumor, and Bcl-2 family plays a major role in this process [25]. As the main members of Bcl-2 family, Bcl-2, Bax and Bcl XL mainly regulate the apoptosis of cells by affecting mitochondrial pathway. When cells get death signals, the Bax which is bonded to Bcl-2 or Bcl-xL will be displaced, results increase in the permeability of the mitochondrial membrane and leading to the release of a series of substances, thus eventually causing the death of cells [26]. Res and PA combination had a strong expression increasing effect of Bax and decreasing effects of Bcl-2, Bcl-xL, Res and PA might raise anticancer effects in HepG2 cancer cells by these mechanisms.

Fas, FasL and caspase-3 are the important proteins mediating the apoptosis of cells. At present, it has been found that FasL could be induced by certain stress responses, such as ultraviolet and DNA damage, and the interactions between FasL and Fas could induce programmed death of cells, which may be an important mechanism of the body to clear cells having mutation [27]. FasL could express on the surface of tumor cells, and tumor-specific antigen could induce tumor infiltrating T lymphocytes (TIL) to express Fas in large quantity, it enhances the sensibility of T cells to apoptosis. Tumor cells induce the apoptosis of T lymphocyte which cause the high expression of Fas by FasL, resulting in immunosuppression. Fas-mediated apoptosis is also related to many other factors, such as p53 gene mutation or the lack of co-stimulatory factor [28]. As cancer related expressions, Fas and FasL also be reduced by treated with Res and PA combination, this combination had a good anticancer effect in HepG2 cancer cells.

p53, the major protein regulating Bcl-2 family, regulates different proteins of Bcl-2 family in various ways, affecting the biological behaviors of pancreatic cancer. p53 can up-regulate Bax and down-regulatie Bcl-2 or Bcl-xL, affecting the apoptosis of cancer cells, and changing the permeability of mitochondria, thus affecting the function of downstream pro-apoptotic genes [29]. As the clumping factor of CDK, low concentrations of tumor suppressor gene p21 positively regulates the function of CDK, facilitating the development of cells and promoting the transition from G1 stage to S stage, but highly expressed p21 protein and cyclin bind to CDK competitively to inhibit the activity of CDK, causing the cell development stagnating in G1 stage, thus inhibiting cell proliferation or inducing cell apoptosis [30]. p73 and p53 protein have homology in target gene binding, but their functions have great differences. As p73 can arrest cell cycle and induce cell apoptosis, it can inhibit tumor to certain extent [31]. Res and PA combination also showed the strong effects on p53 and p21 expressions, these effects might be the important mechanism of Res and PA combination.

Apoptosis inhibiting genes HIAP-1 and HIAP-2 can inhibit caspase to weaken its function to induce apoptosis. Therefore, regulating and weakening the functions of HIAP-1 and HIAP-2 genes is conductive to the activation of caspase, inducing the apoptosis of cancer cells [32].

NF-κB system is composed of NF-κB family and its inhibitor IκB-α. NF-κB is an extremely important transcriptional activator, and IκB-α is the inhibitory protein of NF-κB [33]. NF-κB is important to inflammation process, and also serves as regulatory protein in the development of cancer. It plays an important role in information transmission in relation to tumor growth, closely related to the incidence and development of tumor [34]. Studies have found that NF-κB highly expresses in many types of tumors, and activated NF-κB promotes the expression of a variety of genes which involve the development of cancer [35, 36]. Wu et al. [37] found that Hp infection, activated NF-κB and the expression of COX-2 play important roles in the incidence and development of cancer.

COX-2 and iNOS are not only the target molecules of inflammation, but are also closely associated with the development of tumor, especially colon cancer. The increased expression of COX-2 and iNOS can change signal transduction pathway, leading to the occurrence, invasion and metastasis of tumor [38]. At the same time, iNOS can induce the expression of COX-2, and catalyze the production of NO to enhance the activity of COX-2. It signifies COX-2 and iNOS complement each other to cause cancer. Inhibition of inflammatory factors COX-2 and iNOS with their synthesis can block the proliferation of tumor cells and improve disease, treating colon cancer [39]. IκB-α. NF-κB, COX-2 and iNOS are important cancer related expressions, Res and PA combination showed anticancer effects through raising IκB-α and reducing NF-κB, COX-2, iNOS expressions, and these effects were stronger than only PA treatment.

Malignant tumors are characterized by local invasion and distant metastasis, which are the main reasons that malignant tumor threaten patients’ health and life. MMPs play an important role in the invasion and metastasis of tumor, it not only mediates tumor cells’ degradation of extracellular matrix including the basement membrane, but also controls the process of angiogenesis, that affects the function of cell adhesion molecules and regulates the growth of tumor cells [40]. Study has shown that the expression of MMP-2 and MMP-9 is closely related to cancer angiogenesis; tumor cells which can secrete MMP-2 and MMP-9 have high invasion and metastases ability, drugs can also be used to inhibit the growth of tumor cells through lowering the activity of MMP-2 and MMP-9 [41]. In addition, ECM play a key role in local invasion and distant metastasis of cancer cells, the degradation of ECM is complex, as it involved a lot of factors, and MMPs and inhibitors play important functions, MMPs can degrade ECM, while TIMPs can inhibit the degradation of ECM through lowering the activity of MMPs, to protect normal cells [42]. The formation of intravascular cavity depends on the balance of MMPs and TIMPs, introducing exogenous inhibitors may break the balance of MMPs and TIMPs, inhibiting the process of angiogenesis, as well as the invasion and metastasis of tumor cells. Therefore TIMPs can inhibit tumor invasion and metastasis, and has a remarkable use in the research of tumor treatment [43]. TIMP-1, TIMP-2, MMP-2 and MMP-9 are cancer metastasis related expressions, Res and PA combination could raise TIMP-1, TIMP-2 and reduce MMP-2 and MMP-9 expressions in HepG2 cancer cells, and these effects were stronger than only PA treatment.

EGF is a kind of growth factor which can affect many reactions by combining with EGFR [44]. Study has shown that EGF and other growth factors could promote the proliferation of human cells. EGFR is a member of ErbB receptor family located on the surface of cells, involved in cells proliferation, growth, migration and infiltration [45]. Study has found that EGFR could be adjusted by EGF-meditated cancer cell proliferation through sialylation [46].

VEGF can promote the growth of tumor and angiogenesis, and provides a foundation for tumor metastasis, affecting the prognosis of patients with tumor. VEGF is the strongest vascular endothelial cell growth factor which can directly work on blood vessels, and specifically promote the division, proliferation and migration of endothelial tumor cells, playing an important role in the formation of tumor blood vessel, and it is also one of the key factors of promoting angiogenesis [47]. Fit-1 is the receptor of VEGF, it can bind to VEGF in high affinity. Fit-1 receptor deficient mice are mainly characterized by vascular endothelial cell damage, the expression of Fit-1 is mainly related to the early-stage angiogenesis and wound healing of mouse embryos [48]. PA might inhibit the cancer by decreasing EGF, EGFR, VEGF, Fit-1 expressions, and Res could raise these effects.

High concentrations treatment of paclitaxel and resveratrol have some toxic effects, reducing their therapeutic concentrations could be conducive to cancer treatment [49, 50]. The concentration of paclitaxel and resveratrol combination treatment was lower than the alone treatment. This study showed these effects in vitro, this combination could reduce the usage amount of drug, this is the most important mechanism of action.

## Conclusion

In this in vitro study, there was a deduction that paclitaxel had a good anticancer effect on HepG2 cells, the no toxicity concentration of resveratrol raise and enhance this anticancer effect substantially. Paclitaxel and resveratrol combination treatment could reduce the concentration used alone, this mechanism could reduce drug use concentration and increase the effects. From these results, we can conclude that resveratrol could be used as a sensitizing agent for paclitaxel, and this combination might be use in clinical application to save human life in future.
